# Extraosseous Calcifying Odontogenic Cyst Initially Interpreted as a Parulis

**DOI:** 10.1155/2024/8966953

**Published:** 2024-01-12

**Authors:** Karla Lizeth Santana-Arenas, Israel Guardado-Luevanos, Miguel Padilla-Rosas, Mario Nava-Villalba

**Affiliations:** ^1^Pathology Research and Diagnostic Center, Department of Microbiology and Pathology, University Center of Health Sciences, Universidad de Guadalajara, Guadalajara, Jalisco, Mexico; ^2^Master's Program in Oral Pathology and Medicine, Department of Integral Dental Clinics, University Center of Health Sciences, Universidad de Guadalajara, Guadalajara, Jalisco, Mexico

## Abstract

Extraosseous variants of odontogenic cysts are an infrequent clinical finding, although they have a relatively indolent biological behavior compared to their intraosseous counterparts; due to their nature and clinical appearance, these lesions can be confused with multiple entities that affect soft tissues, so their diagnosis can only be achieved through surgical removal and subsequent histopathological analysis. The extraosseous/peripheral variant of calcifying odontogenic cyst (E/PCOC) has a heterogeneous clinical presentation mainly in terms of size and involvement or not of adjacent anatomical structures such as bone and teeth; in addition, there are few cases reported to date; thus, there are still clinical features to be elucidated. This report presents a child affected by E/PCOC in an unusual location, as well as its therapeutic management, which at first time was suspected of endodontic nature, due to a history of dental trauma.

## 1. Introduction

Calcifying odontogenic cyst (COC) represents ≤1% of all odontogenic cysts, and its extraosseous/peripheral variant (E/PCOC) represents 3-10%, in contrast to the intraosseous/central variant of COC (90-97%), making E/PCOC an unusual lesion [1, 2]. In this sense, to our knowledge, 41 cases of E/PCOC have been reported to date [2–6]. COC was considered as a tumor in the 2005 WHO classification; however, in the later (2017) [7], it was redefined as a simple cyst with ameloblastoma-like epithelium, and in the latest (2022), WHO classification is defined as a “developmental odontogenic cyst characterized histologically by ghost cells, which often calcify” [1], removing the ameloblastic features of epithelium. E/PCOC has a slight tendency to affect the mandible more than maxilla; in both cases, the anterior area is usually more prone to its appearance; in addition, the vestibular mucosa is usually more affected than the palatal mucosa [2, 8]. Only around 27% of E/PCOC cases have been reported in the pediatric and adolescent population [3], and most of them occur in the buccal gingiva [8, 9]. Here, we present a case report of a child affected by E/PCOC located in the palatal mucosa, which at first impression was considered as a parulis, due to a previous history of dental trauma.

## 2. Case Presentation

**Figure 1:**
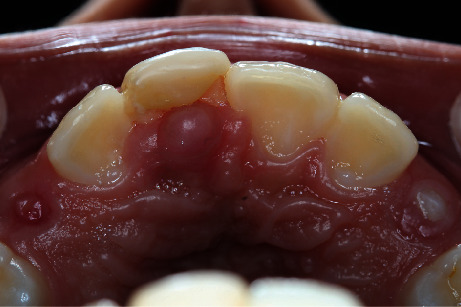
Intraoral photograph, showing a little nodule with an apparently sessile base, located in the palatal mucosa behind the upper right central incisor, noting resin restoration.

**Figure 2:**
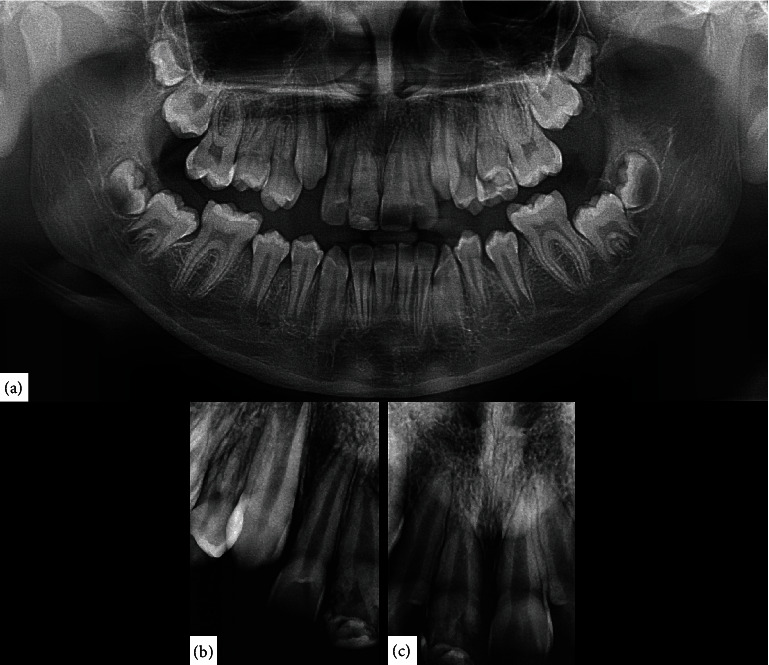
Radiological images. (a) Panoramic X-ray, showing the bone integrity at the level of the upper right central incisor. (b) Periapical radiograph, showing a horizontal coronary fracture of the upper right central incisor can be seen, affecting only enamel and dentin. (c) Periapical radiograph. It can be observed that the length of the tooth is apparently shorter compared with its contralateral counterpart; in addition, the apical region is irregular; however, its formation is complete.

**Figure 3:**
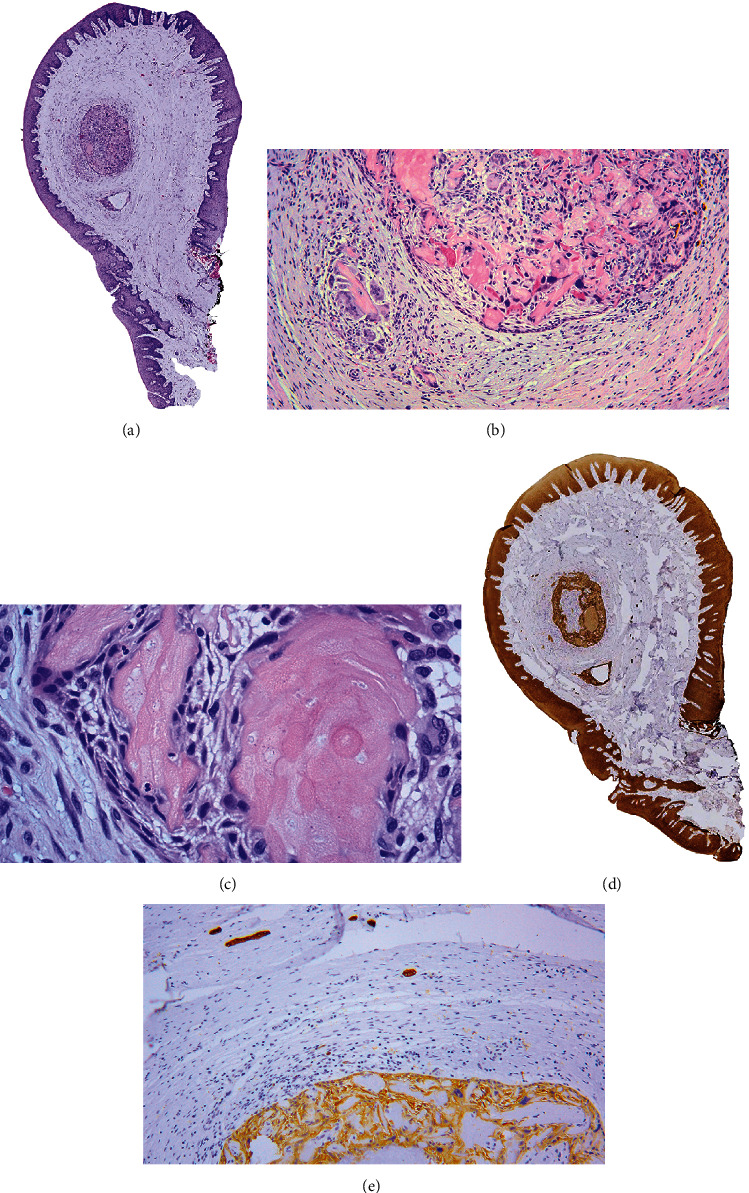
Microphotographs illustrating histopathological findings of the lesion. (a) Panoramic visualization shows a polypoid conformation, one nest, and one cystic-like structure that can be seen in the center of specimen (hematoxylin and eosin (HE) stain; ×20 magnification). (b) Ameloblastoma-like epithelium in the periphery of the nest, dentinoid material, and groups of ghost cells can be seen; additionally, numerous foreign-body type multinucleated giant cells intermixed toward inside and outside in the stroma are seen too (HE stain; ×100 magnification). (c) Conspicuous groups of ghost cells (HE stain; ×400 magnification). (d) CKAE1/AE3 immunoreaction. The luminal space occupied by inflammatory body reaction is negative (×20 magnification). (e) CK19 immunoreaction, cords, and island of odontogenic epithelium surrounding the cystic structures can be identified; it can also be seen in image (d) (×100 magnification).

**Figure 4:**
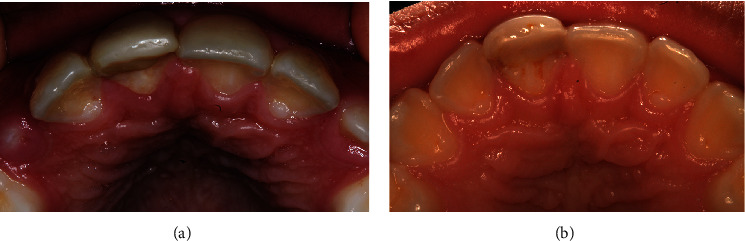
Clinical photographs of follow-up. (a) After three months, a complete recovery is observed. (b) At 4 years of follow-up, no recurrence was observed.

An 11-year-old boy was attended in the Clinic of Oral Pathology and Medicine of the University Center of Health Sciences of the University of Guadalajara, referred by a private practice dentist, due to the presence of a nodule on the palatal mucosa behind the right upper central incisor. During the examination, the patient's mother reported a dental trauma that occurred eight months ago, which caused a crown fracture of the right upper central incisor without exposure of the pulp tissue. Clinical examination revealed a nodule with an apparently sessile base, but narrow in the anteroposterior aspect, with a smooth surface and a slight white color with diffuse posterior erythema (Figure 1). There was no pain on palpation. Panoramic and periapical radiographs were performed, in which no bone involvement was observed (Figure 2). However, due to the history of trauma and clinical impression, an endodontic pathology was needed to be ruled out. He was referred to the endodontic's clinic for evaluation, where thermal sensitivity testing confirmed that the teeth in the area were vital, ruling out an acute or chronic periapical lesion. After ruling out the possibility of an endodontic origin of the lesion, an excisional biopsy was performed under local anesthesia without intraoperative difficulties. Microscopically, a pedunculated mass with two epithelial nests in the center of the specimen was observed, the bigger of them apparently solid, while the minor was cystic, both surrounded by a fibrous stroma in concentric arrangement (Figure 3(a)). The peripheral cells of the nests had ameloblastoma-like features; however, inversed polarization was not typical; and dispersed dentinoid material was also observed (Figure 3(b), Supplementary figure 1-A). Immersed into the epithelial cells resembling the stellate reticulum of the enamel organ, groups of ghost cells were identified (Figure 3(c)). Also, numerous multinucleated foreign body giant cells were found both in the center of the solid nest and outside it and were accompanied by a moderate chronic inflammatory infiltrate (Supplementary figure 1-B). Immunohistochemistry reactions for CKAE1/AE3 and CK19 evidence a central negative zone in the largest nest, confirming the presence of a lumen occupied by the foreign body reaction induced by the ghost cells (Figure 3(d), Supplementary figure 1-C, D). Additionally, the presence of islands and cords of odontogenic epithelium around the cystic epithelium was noted (Figure 3(e), Supplementary figure 1-C). An Ki67 immunoreaction showed a <2% proliferative index in the basal cells of the nests (Supplementary figure 1-E). With all these findings, a diagnosis of E/PCOC with foreign body reaction was made. Seven days after surgery, the child recovered without complications. Subsequently, at 3-month and 4-year follow-ups, no evidence of recurrence was observed (Figure 4).

## 3. Discussion

E/PCOC is an entity that affects principally adult population (73-80%) [3, 8], with an average age of 42 years; interestingly, when it occurs in males, apparently, it presents earlier (33 years) than in females (49 years) [3]. In the young population, the cases are concentrated between seven and twelve years of age or mixed dentition stage [8, 10]; this may be the reason why the identification of E/PCOC is a clinical finding during dental exfoliation or routine dental consultations, as in our case. In general, they are reported as nodules with a sessile base or swellings associated with the vestibular cortex or the crown of an erupting tooth, and there are few cases like the one presented here, with a pedunculated base [8, 11, 12]. In this sense, their clinical presentation is heterogeneous; for example, the average size is 1.1 centimeters [3], but there are cases as large as 7.0 centimeters [13], or as small as ours of a few millimeters [9]. Although it has not been explored whether there is a relationship between size and age, some large cases have been presented in adult patients [13, 14].

Although in the adult population, adult gingival cyst and peripheral odontogenic tumors are the major differential diagnostic considerations; in the young population, reactive lesions seem to be the most recurrent differential diagnostic [4, 8]. In our case, after ruling out possible parulis, the presumptive diagnosis, although uncommon at this age, was oral fibroma, and the diagnosis of E/PCOC was a surprise. It is important to note that even though E/PCOC can present an indolent biological behavior like in our case, sometimes, it can cause root resorption and tooth displacement [8, 12], as well as saucerization/erosion of the underlying bone [8, 15]. Occasionally, bone involvements can only be seen during the surgical procedure [14]. Therefore, because it is not until the histopathological analysis that the nature of the lesion is determined, these cysts continue to be treated by simple excision with or without curettage of the underlying bone, depending on the intrasurgical findings [3]. Low rate of recurrence is notorious in the studies, but a short follow-up of the patients is also noted [3]; thus, it is important to realize that a recurrence has been reported up to seven years after surgery [16].

On the other hand, despite the foreign body reaction being a common feature reported in the intraosseous/central variant of COC, it is an uncommon finding in the E/PCOC [5]. Although it seems a trivial phenomenon, in our case, it represented a challenge, due to the central area of the major element being occupied by this inflammatory reaction, simulating a solid lesion perception; thus, distinction between E/PCOC vs. extraosseous/peripheral dentinogenic ghost cell tumor (E/PDGCT) was only achieved through immunohistochemistry, which allowed us to resolve the topographic expression of cytokeratins in a cystic epithelium conformation. E/PDGCT is the same unusual as E/PCOC, but unlike the intraosseous/central variant of DGCT, which is much more aggressive than COC; E/PDGCT appears to have a very low recurrence potential as does E/PCOC [3, 17]. Because of the above and other clinical aspects, many authors have proposed that it is very likely that extraosseous/peripheral variants of ghost cell lesions are distinct entities from intraosseous/central variants [3, 17, 18].

## 4. Conclusion

The presence of E/PCOC in young patients is not common, and its biological behavior can be quite indolent; therefore, diagnosis and treatment can be delayed. This case complements the existing literature and highlights the importance of periodic visits to the dentist facing any lesion that does not heal or is frankly suspicious regardless of age.

## Data Availability

Data supporting this case report are available from the corresponding author or first author on reasonable request.

## Supplementary Materials

Supplementary Materials Figure S1: additional microphotographs illustrating histopathological findings of the lesion.
