# Supplementary material for: Extraosseous Calcifying Odontogenic Cyst Initially Interpreted as a Parulis
Source: Case Rep Dent. 2024 Jan 12;2024:8966953. doi: 10.1155/2024/8966953 (PMC10798835; doi:10.1155/2024/8966953)
Supplement: Supplementary Materials — Figure S1: additional microphotographs illustrating histopathological findings of the lesion. [file 8966953.f1.docx]

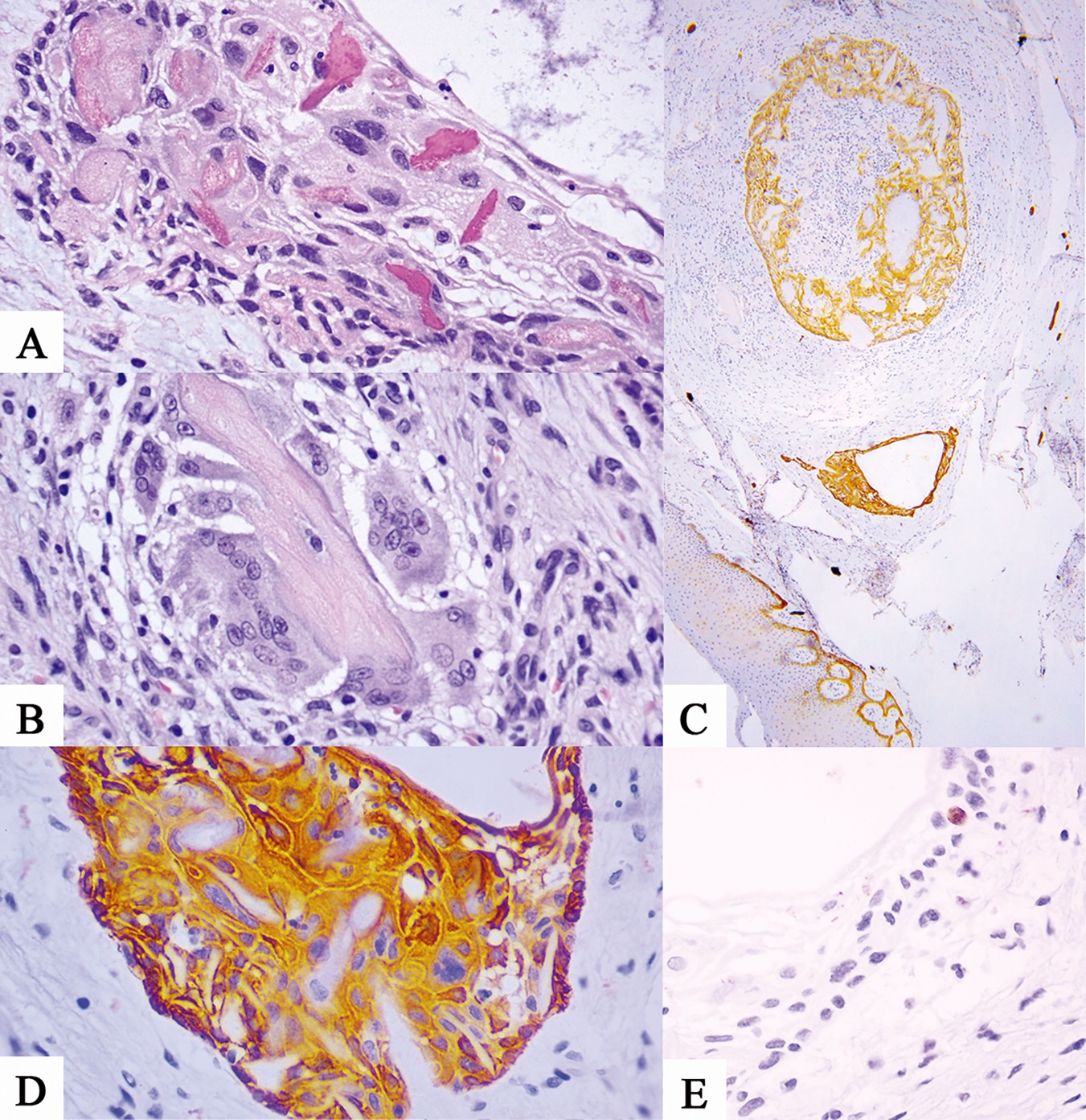


Figure S1. Additional microphotographs illustrating histopathological findings of the lesion. (A) Ameloblastoma-like epithelium in the periphery of the nest, some groups of ghost cells and dentinoid material can be seen in the cystic component of the lesion (hematoxylin & eosin [HE] stain; × 400 magnification). (B) Foreign-body reaction adjacent to the peripheral stroma of the major nest is seen (HE stain; × 400 magnification). (C) Low magnification shown the nest and the cystic structure, note the weak immunolabeling probably due by intense inflammatory reaction in the nest epithelium, basal layer as internal control can be seen in superficial epithelium (CK19 immunoreaction; × 40 magnification). (D) CK19 immunoreaction of the cystic epithelium, the dentinoid material observed in Fig. S1-A is unlabeled (× 400 magnification). (E) Ki-67 immunoreaction (× 400 magnification).
